# Supplementary material for: A qualitative exploration of using financial incentives to improve vaccination uptake via consent form return in female adolescents in London
Source: PLoS One. 2020 Aug 21;15(8):e0237805. doi: 10.1371/journal.pone.0237805 (PMC7446903; doi:10.1371/journal.pone.0237805)
Supplement: S1 File — (PDF) [file pone.0237805.s001.pdf]

Questionnaire items

Front page for girls' questionnaire

UCL banner and coloured pages (different from parents' booklet)

Questionnaire booklet

The HPV vaccination and you

We are researchers from University College London (UCL). We are interested in the HPV vaccine.

This booklet contains some questions about:

- You
- You and your friends
- Returning the HPV vaccine consent form

It should take about 10 minutes to complete.

Remember, **this questionnaire is completely confidential.**

You don't need to write your name on it.

We will NOT show your responses to your parents /guardians or anyone who knows you and your teacher will not look at your answers.

In September you were invited to have the HPV vaccine (jab). This is also known as the cervical cancer vaccine. Your school gave you some information about the HPV vaccination to take home. This information would have included a consent form that you were asked to give to your parent / guardian. You were asked to get your parent / guardian to sign the form and then return it to school. If your parent or guardian said yes to vaccination on this form, you would have been allowed to have the vaccine. If your parent or guardian said no to vaccination on this form, or you did not return the form, you probably would not have been allowed to have the vaccine.

When you were given the HPV vaccine consent form, how motivated were you to get it signed and return it?

Tick one box

Very motivated	Quite motivated	Neither motivated or unmotivated	Quite unmotivated	Very unmotivated
☐	☐	☐	☐	☐

How easy was it for you to remember that you needed to return the consent form?

Tick one box

Very easy	Quite easy	Neither easy or hard	Quite hard	Very hard
☐	☐	☐	☐	☐

We would like you to think about the day that you were given the consent form to take home. When did you give the consent form to your parent / guardian?

Tick ONE box that best describes what happened.

☐ I gave them the form as soon as I/they got home

☐ I gave them the form later that day

☐ I gave it to them the next day

☐ I gave it to them within a couple of days

☐ I gave it to them after about a week

☐ I gave it to them more than a week after getting it

☐ I never gave it to them

Did you see any short-term benefits to returning the HPV vaccine consent form (by 'short-term benefits' we mean good things that might happen in the weeks after you returned the consent form)

☐ Yes   If yes, what did you think were the short-term benefits……………………………………………………………………………..

☐ No

☐ Not sure

How important was it to you to return the consent form?

Tick one box

Very important to me	Quite important to me	Neither important or unimportant	Quite unimportant to me	Very unimportant to me
☐	☐	☐	☐	☐

Did any of these things happen when you were given your HPV vaccine consent form to take home?

Tick all that apply

Yes	No	
☐	☐	I gave my parent / guardian the consent form
☐	☐	I asked my parent / guardian to sign the consent form
☐	☐	My parent gave me the signed consent form to take back to school
☐	☐	I handed the consent form in at school

Next, here are some questions about you and your friends. They are NOT about the HPV vaccine.

Please tell us how true each of these statements are of you. Tick one box for per row.

	Not true of me	Slightly true of me	Moderately true of me	Very true of me	Extremely true of me
I fear others have more rewarding (better) experiences than me	☐	☐	☐	☐	☐
I fear my friends have more rewarding (better) experiences than me	☐	☐	☐	☐	☐
I get worried when I find out my friends are having fun without me	☐	☐	☐	☐	☐
I get anxious when I don't know what my friends are up to	☐	☐	☐	☐	☐
It is important that I understand my friends "in jokes"	☐	☐	☐	☐	☐
Sometimes, I wonder if I spend too much time keeping up with what is going on	☐	☐	☐	☐	☐
It bothers me when I miss an opportunity to meet up with friends	☐	☐	☐	☐	☐
When I have a good time it is important for me to share the details online (e.g. updating my status)	☐	☐	☐	☐	☐
When I miss out on a planned get-together it bothers me (this could be because you were not invited or because you forgot or because you could not go)	☐	☐	☐	☐	☐
When I go on holiday, I continue to keep tabs on what my friends are doing	☐	☐	☐	☐	☐

A few weeks ago you were offered the chance to be entered into a prize draw to win a £50 voucher if you returned the HPV vaccine consent form signed by your parent. All girls were entered into the prize draw if their parent / guardian signed the form. It did not matter if they said yes to vaccination or no to vaccination.

What did you think about being entered into a prize draw to win a £50 voucher if you returned the HPV vaccine consent form? We're interested in your opinions on using a prize draw to encourage girls to bring the form back to school. *(Please write)*

Free text response

In the future, would you return a vaccination consent form if you were not entered into a prize draw to win a £50 voucher?

Yes, definitely	Yes, probably	Not sure	Probably not	Definitely not
☐	☐	☐	☐	☐

Finally, we would like to know a little bit about the people that completed this questionnaire.

What is your religion?

Tick the box that best describes your religion

☐ No religion	☐ Hindu	☐ Sikh
☐ Christian (all denominations, for example Church of England and Catholic)	☐ Jewish	☐ Other......................................
☐ Buddhist	☐ Muslim	

How religious are you?

Tick on the scale to show how religious you feel you are

Not religious at all						Very religious	
1	2	3	4	5	6	7	Don't know
☐	☐	☐	☐	☐	☐	☐	☐

Where were you and your parents born?

Tick the box in each row that best describes where you and each of your parents were born

	Born in the UK	Born outside the UK	Don't know
Me	☐	☐	☐
Mum	☐	☐	☐
Dad	☐	☐	☐

What is the language that you speak most often at home? *(please write)*

Free text response

Is there anything else that you would like to say? *(please write)*

Free text response

Back page of girls' questionnaire

Thank you for taking the time to complete this questionnaire. Please put it in the envelope that came with it and return it to your teacher, who will give it to the researchers.
