# Supplementary material for: A qualitative exploration of using financial incentives to improve vaccination uptake via consent form return in female adolescents in London
Source: PLoS One. 2020 Aug 21;15(8):e0237805. doi: 10.1371/journal.pone.0237805 (PMC7446903; doi:10.1371/journal.pone.0237805)
Supplement: Attachment — Submitted filename: Response to reviewer comments.docx [file pone.0237805.s002.docx]

**PLoS ONE response to reviewer comments**

**Editor**

| **Editor comments** | **Response to editor comments and changes made** |
| --- | --- |
| Firstly, the manuscript title does not adequately reflect the study background and should be reworded. The abstract should include some important demographic variables, i.e. setting of study, age range of the participants, and specification regarding female secondary students (instead of “adolescents”). | We have now provided this information in the title and abstract, in line with the comments provided by Reviewer 1. |
| The Introduction section is should be shortened; some information on vaccine coverage in the country/at the study site should be provided, together with quantification of the problem caused by non-return of consent forms. | We have removed the lines suggested by Reviewer 2 and sought to shorten the introduction further elsewhere. We have retained our discussion of the possible plausible mechanisms of action for incentives. We know of no similar summary, and feel it would be useful to the research community to include. |
| Sampling criteria should be thoroughly described, possibly with the help of graph. Table of demographics for the two studies should be included. | We have provided further detail on the sampling criteria, namely how schools were selected for participation. We now also provide a table of the demographic characteristics of the samples. |
| More detailed information of the rest of the cohort would be of value. | We have now included, in the demographic characteristics table, detail of the overall cohort from where the samples for this study were taken. This includes vaccination status. |
| References are presented in a sloppy way, this should be changed following the journal criteria. | We have reviewed and amended the references. |
| Please ensure that your manuscript meets PLOS ONE's style requirements, including those for file naming. The PLOS ONE style templates can be found at  https://journals.plos.org/plosone/s/file?id=wjVg/PLOSOne_formatting_sample_main_body.pdf and  https://journals.plos.org/plosone/s/file?id=ba62/PLOSOne_formatting_sample_title_authors_affiliations.pdf | We have amended the manuscript accordingly. |
| Please include additional information regarding the survey or questionnaire used in the study and ensure that you have provided sufficient details that others could replicate the analyses. For instance, if you developed a questionnaire as part of this study and it is not under a copyright more restrictive than CC-BY, please include a copy, in both the original language and English, as Supporting Information | We have now provided the questionnaire used for Study 2 as Supporting Informationl. |
| We note that you have indicated that data from this study are available upon request. PLOS only allows data to be available upon request if there are legal or ethical restrictions on sharing data publicly. For more information on unacceptable data access restrictions, please see http://journals.plos.org/plosone/s/data-availability#loc-unacceptable-data-access-restrictions. | Data are available at https://rdr.ucl.ac.uk/articles/dataset/Acceptability_of_incentives/12659792 |

**Reviewer 1**

| **Reviewer 1 comments** | **Response to Reviewer 1 comments and changes made** |
| --- | --- |
| Thank you very much for allowing me the opportunity to review the manuscript titled "A qualitative exploration of using financial incentives to improve vaccination uptake in adolescents" (PONE-D-20-13180). I felt confident that the authors performed careful and thorough analysis of the interesting data, however I feel like the manuscript has some errors that make it more difficult to read. I have a few comments and questions in regard to the paper; therefore I recommend that a minor revision is warranted. Please see some specific comments below. | - |
| Title: Authors should be more specific in title – including that the participant cohort were only female and the setting took place in London, UK. Moreover, throughout the whole manuscript, it was emphasized that incentives were used to improve consent form return rate. “A qualitative exploration of using financial incentives to improve vaccination consent form return rate in female adolescents in London, UK.” | We have added that participants were female, that the study was conducted in London and that the study was looking at incentives to improve vaccine uptake via consent form return. |
| Abstract: Include setting of study, age range of the participants, and specify they are female secondary students instead of “adolescents” for both study 1 and 2 under methods section. | We have added that the participants were female secondary students, with their ages specified and that the studies took place in London. |
| Introduction: Line 52 – It would be informative for readers if the decline of uptake in England was quantified | Since 2012/2013 England has seen consistent declines in uptake for most routine infant vaccines, with drops in the region of 2-3 percentage points. We have added this to the manuscript. |
| Line 53 – similarly, it would also be informative for readers if the size of the 2018 measles outbreak was also quantified – how many were ill? | There were 989 confirmed cases of measles in 2018. This has been added. |
| Line 56 – Would be clearer to read if “In the UK programmes” was clarified to “In school-based vaccination programmes in the UK...” | We have made this change. |
| Line 58 - Authors said “consent forms must be returned” but in next sentence, it is said that “large proportions of consent form are not returned” which is contradicting, I would rephrasing those two sentences. It would be helpful to have confirmed that unreturned consent forms are considered consent declined. | We have rephrased the first sentence to reflect the fact that parents are asked to indicate on the consent form whether they are giving or declining consent to vaccination. We also note that vaccinations will not be given in most instances where consent forms are unreturned. |
| Line 73 – extra parentheses after 8, 9 citations | This has been removed. |
| Line 73 – “Most commonly they are operationalised as the offer of a reward by someone other than the target individual, although individuals may self-incentivise” It would be helpful for understanding of this sentence by providing readers of example. | We have now removed this sentence to shorten the introduction, as requested by Reviewer 2. |
| Line 93 – within the cited study, was the 76% compared to 61% vaccine uptake in cohorts found as a statistically significant difference? | It was not appropriate to test for significance in this feasibility study, a larger study is required to explore efficacy. We have added this information. |
| Line 96 – include “vaccination” between “Hepatitis B” and “consent forms” | We have made this change. |
| Line 149 – change to “the objectives of the paper are” as there are two objectives | We have made this change. |
| Methods and Materials  Line 159 – “In brief, participants were six secondary schools in two London boroughs” is not clear | For the original cluster randomised controlled trial, participants were schools (as the trial was a cluster design). We acknowledge this is confusing in the context of the present paper and have revised the sentence. |
| Line 258 – “themes in Study and 2” unclear | We have corrected this error. |
| Line 263 – Either bring up Table 1 closer to paragraph end or specify that Table 1 in Results section | We have specified that Table 1 (now Table 2) is in the results section. |
| Results  It would be informative to have a table of demographics for the two studies. For example, line 274 mentions 37% of whom reported to be White British ethnic background. It would be informative for readers to have more detail of the rest of the cohort, as well as the other information collected like religion, birthplace and vaccination uptake within the previous feasibility study. | We have now included a table of demographic characteristics of the sample for both studies and the previous trial. |
| Line 278 – “eight had received no doses of the vaccine” does this mean that they have declined consent to be vaccinated? | It is difficult to know why these individuals had received no doses of the vaccine. They or their parents may have actively declined consent, or they may have not been present at the school on the day of vaccination (even though their parents had consented to vaccination). |
| Line 324 – would not consider “commonly expressed” with 18% of responses | We have revised this sentence (‘this was also expressed by respondents…’). |
| Table 1 – It would be useful to have a more specific table title, in case the table would stand alone. Example, to add this is from Study 2. | We have now provided a more detailed Table title. |
| Table 2 – Would also be useful to specify that this is for Study 1 | We have now detailed that Table 2 (now Table 3) refers to Study 1. |
| Line 369 – Do not understand in quotation “Get for a vaccination” | We agree that this was confusing and not adding to the quote. We have removed the sentence. |
| Table 3 – add in title from Study 1 quotes and Study 2 free text responses | We have made Table 3 (now Table 4) clearer, explaining where the quotes and frequencies come from. |
| Discussion  Line 524 – important that authors have noted that most participants in study were from families with low levels of socio-economic disadvantage – would be interesting to have study of a mixed cohort population and compare findings | This would be an interesting comparison. We have added this to our discussion. |
| References  Authors should review references list to standardize the way journal names – in some, journal names are all capitalised, some only sentence case, and some have short form of the journal | We have reviewed and amended the references. |

**Reviewer 2**

| **Reviewer 2 comments** | **Response to Reviewer 2 comments and changes made** |
| --- | --- |
| Reviewer #2: This is a well written manuscript that is easy to follow. I’m not expert in qualitative research, but the reporting is clear and framed by the COREQ recommendations. | - |
| Abstract:  Mention that the studies included 12-13 and 13-14 year old girls in London | We have added this information. |
| Introduction:  Well written and informative, but appears to be too long. L 49-53 could be deleted to shorten the introduction, as the information is quite/too general | We have removed the lines suggested by the Reviewer and sought to shorten the introduction further elsewhere. We have retained our discussion of the possible plausible mechanisms of action for incentives. We know of no similar summary, and feel it would be useful to the research community to include. |
| On the other hand, some information on vaccine coverage in the UK / in London / in the included neighbourhoods would be helpful, and some quantification of the problem caused by non-return of consent forms. | We have added the findings of unpublished data that describes that 45% of child flu vaccine consent forms are unreturned to the introduction. We have added vaccine coverage information about the included neighborhoods in the Study Context section of the Methods. |
| Methods: Please describe how schools were selected for participation | We approached all secondary schools in participating boroughs initially via email and then by telephone. We stopped recruitment once a sufficient number of schools agreed to participate. We have added this detail to the manuscript. |
| Line 258: drop “and” | We have made this change. |
| Results: Line 284: “The most commonly reported religion was Christianity (50%) “=> rather say “Christianity was reported by half” | We have made this change. |
| Was there any information about who is responsible for the form not being returned: is this really the adolescents’ choice (which the verbatims seems to suggest), or do the parents have a substantial contribution to the fact that the form is not returned (refusal to sign, loss, etc). | We only have the adolescents’ view on this issue in this study, but it is quite reasonable to assume that both the adolescent and parent have a role to play in returning the form. However, we cannot state this with any confidence based on the findings of our study. |
| Discussion:  Is there evidence that the acceptability is similar among parents (incentive to adolescents)? | We do not have any quantitative data on parents’ acceptability of incentives for improving consent form return, but they do raise similar concerns and discuss similar benefits to the adolescents in our study. We have added this detail to the discussion. |
| Which are the alternatives to incentivisation? Could the schools make the return of forms mandatory, just as other forms must be returned? | Presently, the return of consent form is equally as mandatory as the return of other forms in schools. On a school-by-school basis, young people may receive punishment for not returning forms (a form of disincentive). Immunisation nurses also spend a large amount of time chasing adolescents and their parents to encourage them to return their consent forms. We have added a discussion of this in the introduction. |
